# Accessibility of ENaC extracellular domain central core residues

**DOI:** 10.1016/j.jbc.2022.101860

**Published:** 2022-03-23

**Authors:** Lei Zhang, Xueqi Wang, Jingxin Chen, Thomas R. Kleyman, Shaohu Sheng

**Affiliations:** 1 Departments of Medicine, University of Pittsburgh, Pittsburgh, Pennsylvania, USA; 2 Department of Nephrology, Hunan Key Laboratory of Kidney Disease and Blood Purification, The Second Xiangya Hospital, Central South University, Changsha, Hunan, China; 3 The Third Xiangya Hospital of Central South University, Changsha, Hunan, China; 4 Cell Biology, University of Pittsburgh, Pittsburgh, Pennsylvania, USA; 5 Pharmacology and Chemical Biology, University of Pittsburgh, Pittsburgh, Pennsylvania, USA

**Keywords:** epithelial sodium channel (ENaC), acid-sensing ion channel (ASIC), protein domain, allosteric regulation, homology modeling, channel gating, Na^+^ self-inhibition, extracellular domain, two-electrode voltage clamp, amiloride, ACIC, acid-sensing ion channel, ENaC, epithelial Na^+^ channel, MTS, methanethiosulfonate, MTSES, sodium (2-sulfonatoethyl) methanethiosulfonate, MTSET, [2-(trimethylammonium) ethyl] methanethiosulfonate bromide.

## Abstract

The epithelial Na^+^ channel (ENaC)/degenerin family has a similar extracellular architecture, where specific regulatory factors interact and alter channel gating behavior. The extracellular palm domain serves as a key link to the channel pore. In this study, we used cysteine-scanning mutagenesis to assess the functional effects of Cys-modifying reagents on palm domain β10 strand residues in mouse ENaC. Of the 13 ENaC α subunit mutants with Cys substitutions examined, only mutants at sites in the proximal region of β10 exhibited changes in channel activity in response to methanethiosulfonate reagents. Additionally, Cys substitutions at three proximal sites of β and γ subunit β10 strands also rendered mutant channels methanethiosulfonate-responsive. Moreover, multiple Cys mutants were activated by low concentrations of thiophilic Cd^2+^. Using the Na^+^ self-inhibition response to assess ENaC gating behavior, we identified four α, two β, and two γ subunit β10 strand mutations that changed the Na^+^ self-inhibition response. Our results suggest that the proximal regions of β10 strands in all three subunits are accessible to small aqueous compounds and Cd^2+^ and have a role in modulating ENaC gating. These results are consistent with a structural model of mouse ENaC that predicts the presence of aqueous tunnels adjacent to the proximal part of β10 and with previously resolved structures of a related family member where palm domain structural transitions were observed with channels in an open or closed state.

Epithelial Na^+^ channels (ENaCs) are members of the ENaC/degenerin family of ion channels that are formed by subunits with large extracellular domains that interact with factors in the external environment that regulate channel gating (1, 2). Channel subunits are similar in structure, with complex highly folded extracellular regions that connect to two transmembrane α helices that form the channel pore where the selectivity filter and gate reside and cytoplasmic N- and C-termini (3). ENaCs are heterotrimers composed of α (or δ), β, and γ subunits. They are expressed in the apical membrane of specific cells in the aldosterone-sensitive distal nephron, where the channel has a key role in the reabsorption of filtered Na^+^ and regulation of extracellular fluid volume and blood pressure, as well as facilitating the secretion of K^+^ (1, 2, 4). They are expressed at other sites that also influence blood pressure, including lingula epithelia, monocytes, endothelium, and vascular smooth muscle (4, 5).

The resolved structures of the extracellular regions of an acid-sensing ion channel 1 (ASIC1) and ENaC have provided important insights regarding channel structure and regulation (6, 7, 8, 9, 10). The extracellular regions of members of this ion channel family are formed by five distinct domains, referred to as palm, β-ball, finger, thumb, and knuckle. The palm and β-ball domains are formed by β strands. The more peripheral finger, thumb, and knuckle domains are formed by α helices. ENaCs are regulated by a number of extracellular factors, including Na^+^, H^+^, Cl^-^, proteases, and shear stress (1, 2, 11, 12, 13, 14, 15, 16). For example, extracellular Na^+^ inhibits ENaC by binding to a site in the finger domain of the α subunit, leading to structural transitions that are transmitted to the transmembrane domains with a reduction in channel open probability (10, 17). Channels with a δ subunit from specific species are also inhibited by extracellular Na^+^ (18). Specific proteases cleave the α and γ subunits at multiple specific sites in their finger domains, releasing imbedded inhibitory tracts and transitioning channels to higher open probability states (1, 2). While studies have implicated specific residues and extracellular domain structures in regulating ENaC gating, a clear understanding of the structural transitions that occur in association with gating is lacking.

The palm domains within ENaC subunits are composed of a series of antiparallel beta strands and provide the key link between the extracellular and transmembrane regions of the channel (9, 10). Recent studies suggest that the palm domain has an important role in regulating the gating of members of the ENaC/degenerin family of ion channels (7, 19, 20, 21), although exact mechanisms are unclear. Within the palm domain, a long β strand (β10) resides within the trimeric symmetry axis, bordering the knuckle and thumb domains. β10 residues that are proximal to the channel pore, together with other palm domain β strands, enclose a central vestibule whose function is also unknown (9, 10). To explore the accessibility and potential functional roles of the mouse α subunit β10 strand, individual residues within β10 were systematically mutated to Cys. We also mutated specific residues in the β10 strand of the β and γ subunits to Cys. We examined the functional response of wildtype and mutant channels to the Cys-reactive reagents sodium (2-sulfonatoethyl) methanethiosulfonate (MTSES) and [2-(trimethylammonium) ethyl] methanethiosulfonate bromide (MTSET), as well as the inhibitory response to extracellular Na^+^. We found that channels with specific Cys substitutions in the proximal aspects of the β10 strand of the α, β, and γ subunits are modified by methanethiosulfonate (MTS) reagents, respond to Cd^2+^, and have an altered Na^+^ self-inhibition response, suggesting a role in modulating ENaC gating.

## Results

**Figure 1:**
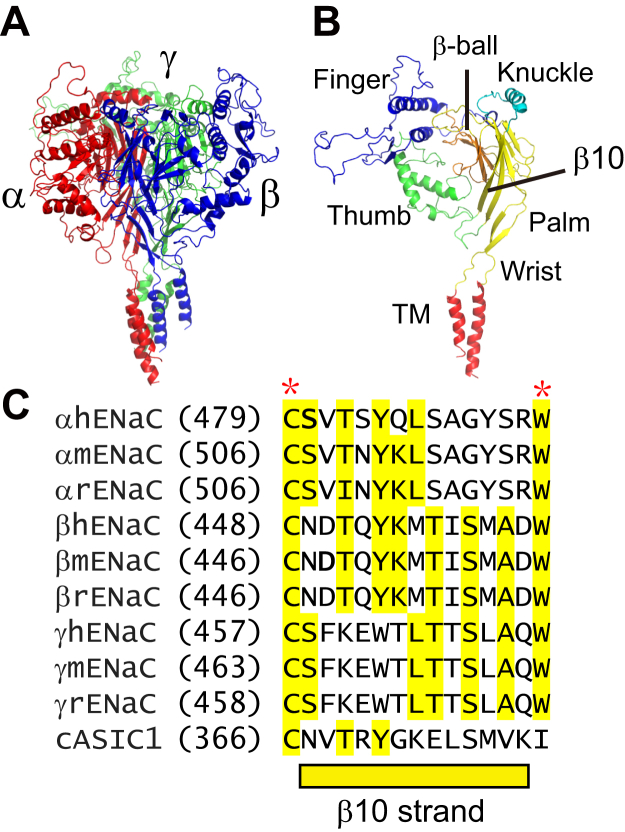
**ENaC model and the β10 strand**. *A*, structural model of mouse ENaC. A structural model of mouse ENaC was generated with SWISS-MODEL, using a trimeric human ENaC structure (PDB 6BQN) and PyMOL 2.4 (75). The α, β, and γ subunits are presented in *red*, *blue*, and *green*, respectively. *B*, structural model of the α subunit. Finger, knuckle, thumb, palm, and β-ball domains are presented in *blue*, *cyan*, *green*, *yellow*, and *orange*, respectively. The transmembrane (TM) α helices are highlighted in *red*. *C*, sequence alignment of human, mouse, and rat ENaC α, β, and γ subunit β10 strands and chicken ASIC1 β10 strand. Identical residues are highlighted. The asterisks identify human α subunit residues where variants associated with a gain-of-function reside (56, 57, 58). Alignments were generated using Clustal Omega (76). ASIC, acid-sensing ion channel; ENaC, epithelial Na^+^ channel.

A structural homology model of mouse ENaC was built using SWISS-MODEL (22), based on the resolved structure of human ENaC (PDB 6BQN (9)). Trimeric and monomeric structural models are shown in Figure 1. The proximal end of β10 links to the thumb domain, whereas the distal end of β10 links to the knuckle domain (Fig. 1*B*). The β10 sequence is well conserved among α, β, and γ subunits of human, mouse and rat ENaC, and chicken ASIC1 (Fig. 1*C*).

Thirteen β10 residues in the mouse ENaC α subunit were individually mutated to Cys. Wildtype and mutant ENaCs were expressed in *Xenopus* oocytes. Amiloride (10 μM)-sensitive Na^+^ currents were monitored by two-electrode voltage clamp, and the response to extracellular MTS reagents (MTSES and MTSET) was assessed. We chose these Cys-reactive reagents as they are similar in size. While MTSES modification adds a negative charge, MTSET adds a positive charge. If a Cys-containing mutant channel responded to either reagent with a significant change in channel current, this Cys was considered as functionally accessible and important. If a Cys mutant channel did not respond to any reagent, this Cys was described as functionally inaccessible. The latter category includes channels with Cys substitutions that were not chemically modified by MTS reagents as well as channels where MTS modifications did not alter activity.

ENaCs are known to be inhibited by extracellular Na^+^, a process referred to as Na^+^ self-inhibition (1). When an ENaC-expressing oocyte is bathed in a solution with a low (1 mM) [Na^+^] at a holding potential of −100 mV, there is negligible inward Na^+^ current. A transition to a high (110 mM) [Na^+^] results in a rapid increase in inward Na^+^ current, which reaches a peak (Ipeak) that is followed by a fall in current, reflecting a reduction in channel open probability due to Na^+^ self-inhibition (11, 12, 23). The Na^+^ current eventually reaches a new steady-state level (Iss), and the ratio of Iss to Ipeak reflects the magnitude of the Na^+^ self-inhibition response. Based on previous studies showing that extracellular cues often regulate ENaC gating by altering Na^+^ self-inhibition response (12, 13, 14, 24), we examined whether MTS reagents modified the Na^+^ self-inhibition response of the Cys mutants that showed a significant change in current in response to these reagents.

### Specific introduced Cys residues in the proximal part of the *α* subunit β10 strand are functionally accessible to MTS reagents

**Figure 2:**
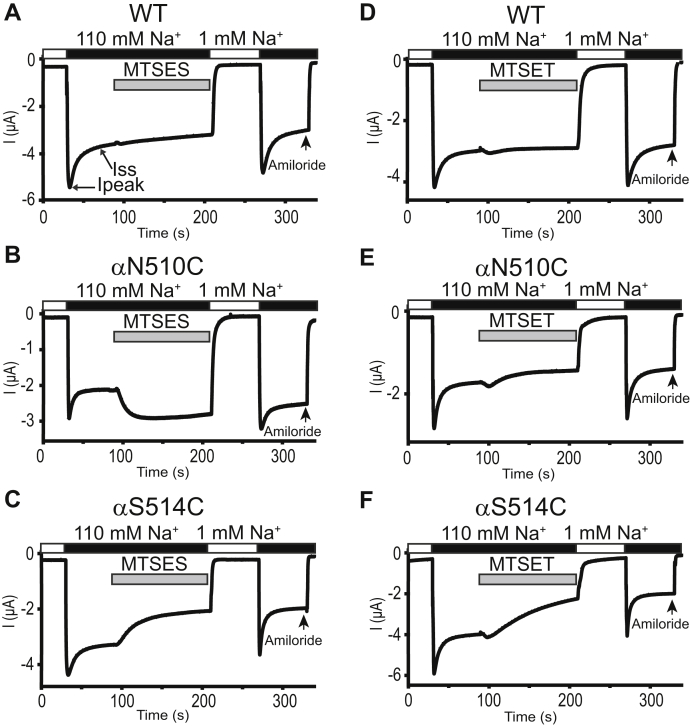
**Select α subunit β10 residues are functionally accessible to MTSES and MTSET**. Oocytes expressing wildtype or mutant mouse ENaC channels were clamped at a holding potential of −100 mV. Whole-cell Na^+^ currents were measured in oocytes perfused with solutions containing either a 1 mM (*white bar*) or a 110 mM Na^+^ (*black bar*), as noted. *A*–*C*, representative recordings of wildtype (WT), αN510Cβγ, and αS514Cβγ channels, respectively, showing the effects of 2 mM MTSES, added to the bath as indicated by the *gray bar*. Ipeak, peak current after switching from a 1 mM to a 110 mM Na^+^ bath solution. Iss, steady state current at 40 s after Ipeak. *D*–*F*, representative recordings of wildtype, αN510Cβγ, and αS514Cβγ channels, respectively, showing the effects of 1 mM MTSET, added to the bath as indicated by the *gray bar*. Arrow indicates addition of amiloride (10 μM) to the bath. MTSES, sodium (2-sulfonatoethyl) methanethiosulfonate; MTSET, [2-(trimethylammonium) ethyl] methanethiosulfonate bromide.

**Figure 3:**
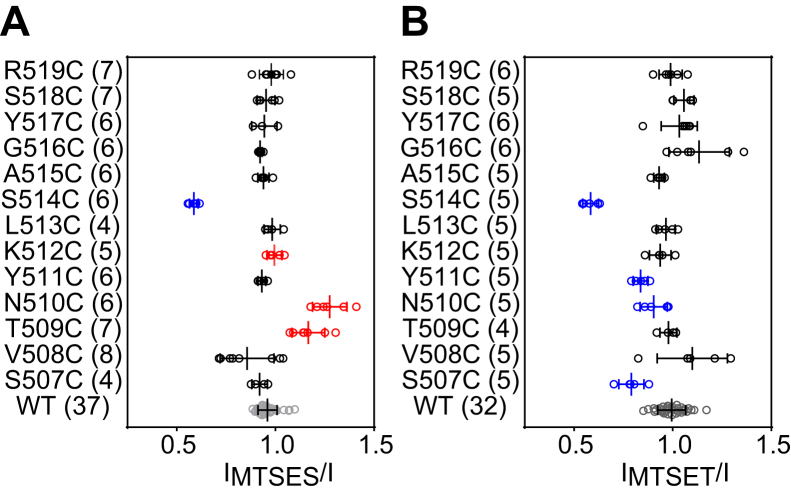
**External MTSES and MTSET alter the activity of multiple α subunit β10 Cys mutants**. *A and B*, scatterplots of I_MTSES_/I and I_MTSET_/I, measured in channels with α subunit β10 Cys mutants. I_MTSES_/I and I_MTSET_/I are ratios of amiloride-sensitive currents after (I_MTSES_ or I_MTSET_) and prior to (I) application of the reagents. Bars are mean ± S.D. Numbers of oocytes in each experiment are listed in parentheses. Wildtype mouse αβγ ENaCs were always examined and compared with the mutant channels in the same batch of oocytes. Statistically significant differences in the MTS response of wildtype and mutant channels were determined in the same batches of oocytes by one-way ANOVA followed by Dunnett’s tests. The values for wildtype channels (WT, shown in *gray*) in the charts were from all batches of oocytes used in these experiments and are shown for display purpose only. Values that are not significantly different from that measured with wildtype ENaC are shown in black. Values that are significantly reduced or increased when compared with wildtype (*p* < 0.05) are shown in blue or red, respectively. ENaC, epithelial Na^+^ channel; MTS, methanethiosulfonate; MTSES, sodium (2-sulfonatoethyl) methanethiosulfonate; MTSET, [2-(trimethylammonium) ethyl] methanethiosulfonate bromide.

All mutant ENaCs expressed sufficient amiloride-sensitive currents to permit examination of their response to externally applied MTS reagents. Similar effects of the MTS reagents were noted when examined before or after assessing the Na^+^ self-inhibition response. Neither MTSES nor MTSET altered currents in oocytes expressing wildtype ENaC, in agreement with previous observations (25, 26) (Fig. 2, *A* and *D*). In contrast, three mutants responded to MTSES with significant increases in whole-cell current. Amiloride-sensitive Na^+^ currents measured in the presence of MTSES (I_MTSES_) were normalized to currents measured prior to the addition of MTSES (I). We observed significant increases in currents in oocytes expressing αT509Cβγ and αN510Cβγ (Figs. 2 and 3*A*), while a very modest but statistically significant increase in I_MTSES_/I was observed with αK512Cβγ (Fig. 3*A*). In contrast, MTSES significantly inhibited αS514Cβγ (Figs. 2*C* and 3*A*). For other Cys mutants, responses to MTSES were similar to wildtype ENaC (Fig. 3*A*).

Four of the 13 α subunit β10 mutants (αS507C, αN510C, αY511C, and αS514C) exhibited significant reductions in amiloride-sensitive Na^+^ currents after application of MTSET (Figs. 2 and 3*B*). For other mutants, responses to MTSET were similar to wildtype ENaC (Fig. 3*B*). The effects on currents in oocytes expressing ENaC mutants were sustained after the reagent was washed out, consistent with irreversible channel modification.

### Modification of specific β10 Cys-substituted residues with MTS reagents alters the Na^+^ self-inhibition response

**Figure 4:**
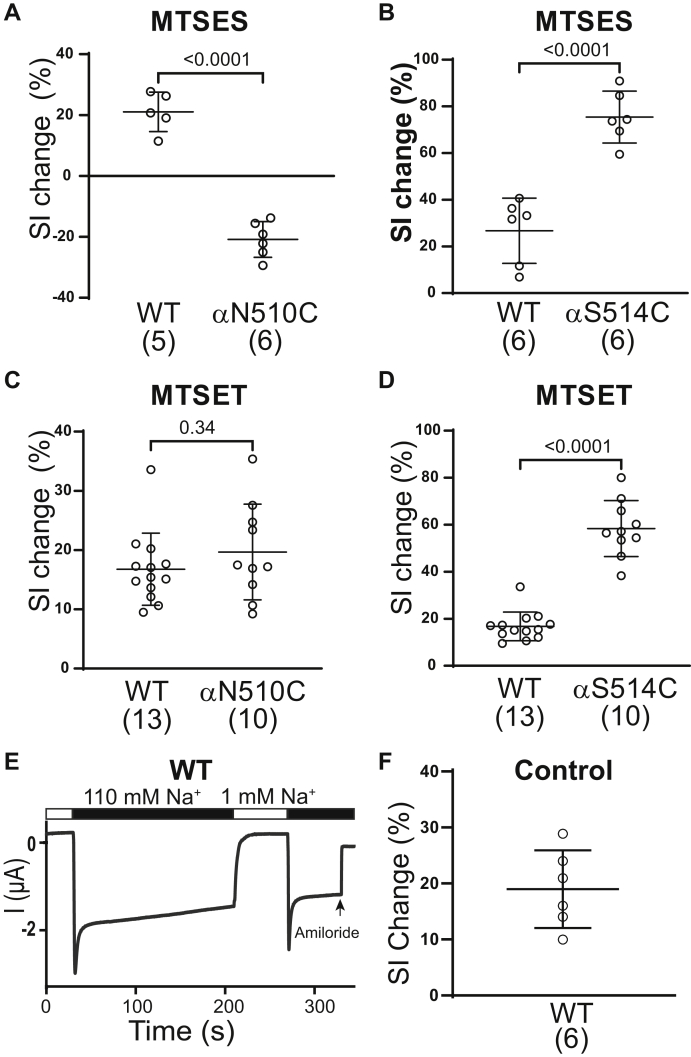
**Effects of MTSES and MTSET on Na**^**+**^**self-inhibition**. Percentage change of the Na^+^ self-inhibition response following MTSES (*A* and *B*) or MTSET (*C* and *D*) treatment were calculated from the following formula: (SI_2_-SI_1_)/SI_1_, where SI_2_ reflects the percentage of the Na^+^ self-inhibition response after MTSES or MTSET [100×(Iss-Ipeak)/Ipeak] and SI_1_ reflects the percentage of the Na^+^ self-inhibition response prior to MTSES or MTSET treatment [100×(Iss-Ipeak)/Ipeak]. Data were from experiments shown in Figure 2. Bars are mean ± S.D., and *p* values were from unpaired *Student’s t* test with Welch’s correction. *E*, representative recording of an oocyte expressing wildtype ENaC showing repetitive Na^+^ self-inhibition responses (n = 6). The experiment had the same time course as in Figure 2, *A* and *D*, except that no MTSES or MTSET was applied. *F*, Na^+^ self-inhibition change (%) from experiments illustrated in (*E*). Note the similar values to that in (A-D) for wildtype. MTSES, sodium (2-sulfonatoethyl) methanethiosulfonate; MTSET, [2-(trimethylammonium) ethyl] methanethiosulfonate bromide.

We examined the Na^+^ self-inhibition response of wildtype and mutant channels that showed significant changes in current in response to MTS reagents, by measuring the Iss to Ipeak ratio following the switch from a 1 mM to 110 mM Na^+^ bath (see Fig. 2*A*). The Na^+^ self-inhibition response was determined prior to and following MTSES or MTSET application. The percent change in the Na^+^ self-inhibition response (ΔSI) following MTS treatment, relative to the Na^+^ self-inhibition response prior to MTS, was determined using the formula described in the Figure 4 legend. A positive ΔSI reflects an enhanced Na^+^ self-inhibition response after MTSES or MTSET treatment, while negative ΔSI reflects a reduced Na^+^ self-inhibition response after MTSES or MTSET treatment. Wildtype ENaCs showed a similar and modest increase in Na^+^ self-inhibition after MTSES and MTSET treatment (Fig. 4, *A*–*D*). This increase is likely not due to the MTS reagent, as we have observed that repetitive tests of Na^+^ self-inhibition in the same oocyte often led to a modest increase in the Na^+^ self-inhibition response. To ascertain this was the case, we examined Na^+^ self-inhibition responses in oocytes expressing wildtype ENaCs twice with the same time interval as the MTS application shown in Figure 2, *A* and *D* (*i.e.*, 2 min). The representative recording and summary data are shown in Figure 4, *E* and *F*, demonstrating a similar level of increase in the Na^+^ self-inhibition response of wildtype channels as observed after MTS application (Fig. 4, *A*–*D*). This increase in Na^+^ self-inhibition may be related to a time-dependent decrease in ENaC activity that has been observed when oocytes are bathed with a high concentration of extracellular Na^+^ and clamped at hyperpolarization potential (27, 28). Compared to wildtype, MTSES significantly reduced the Na^+^ self-inhibition response of αN510Cβγ but increased that of αS514Cβγ. MTSET, on the other hand, did not significantly alter the Na^+^ self-inhibition response of αN510Cβγ but increased the Na^+^ self-inhibition response of αS514Cβγ. These results suggest that MTSES and MTSET modify the Na^+^ self-inhibition response of specific mutant channels.

### MTSES does not activate αN510C channels containing a βS518K mutation

**Figure 5:**
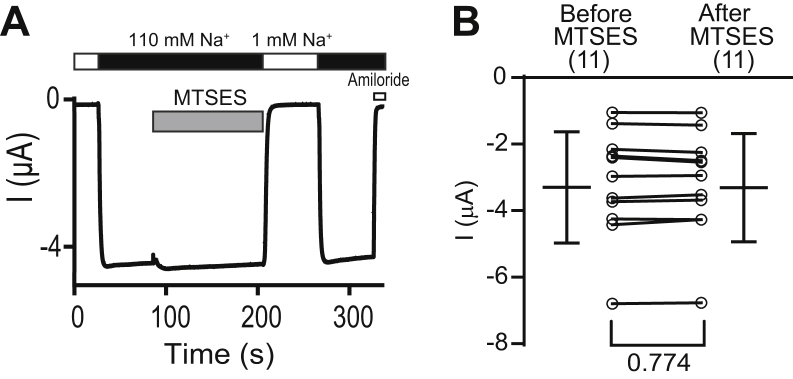
**MTSES activation of αN510C mutant is absent in channels containing the βS518K mutant**. *A*, representative recording showing the effect of MTSES on αN510CβS518Kγ. Oocytes were clamped at −100 mV and perfused with either a 1 mM or a 110 mM Na^+^ bath solution, as indicated by *white* and *black* bars, respectively. MTSES (2 mM) was applied for 2 min as indicated by the *gray* bar. Amiloride (10 μM, open bar) was applied at the end of the experiment. Representative of 11 experiments. *B*, amiloride-sensitive currents before and after MTSES treatment are shown as circles connected by lines. Similar currents were observed prior to MTSES (−3.2 ± 1.6 μA) and after MTSES (−3.2 ± 1.6 μA, n = 11, *p* = 0.774 from paired *Student t* test) treatment. Bars on the *left* and *right* are mean ± S.D. before and after MTSES treatment, respectively. MTSES, sodium (2-sulfonatoethyl) methanethiosulfonate.

The strong activation of αN510Cβγ by MTSES was associated with a significant reduction in the Na^+^ self-inhibition response (Figs. 2*B* and 3*A*), consistent with an MTSES-dependent increase in channel open probability in the presence of a high extracellular [Na^+^]. An increase in current could also reflect an increase in unitary channel conductance. To rule out this possibility, we examined the effect of MTSES on αN510C channels that also had a βS518K mutation. Previous studies have shown that βS518K channels have an intrinsic high open probability (29, 30, 31). If MTSES activation involves an increase in unitary conductance, it should activate αN510CβS518Kγ channels. We observed that MTSES did not activate αN510CβS518Kγ (*p* = 0.77, before *versus* after MTSES treatment, n = 11, paired Student’s *t* test, Fig. 5). Consistent with a high open probability state, the Na^+^ self-inhibition response of αN510CβS518Kγ was absent prior to and after MTSES application (Fig. 5*A*).

### Four α subunit β10 mutations alter Na^+^ self-inhibition response

**Figure 6:**
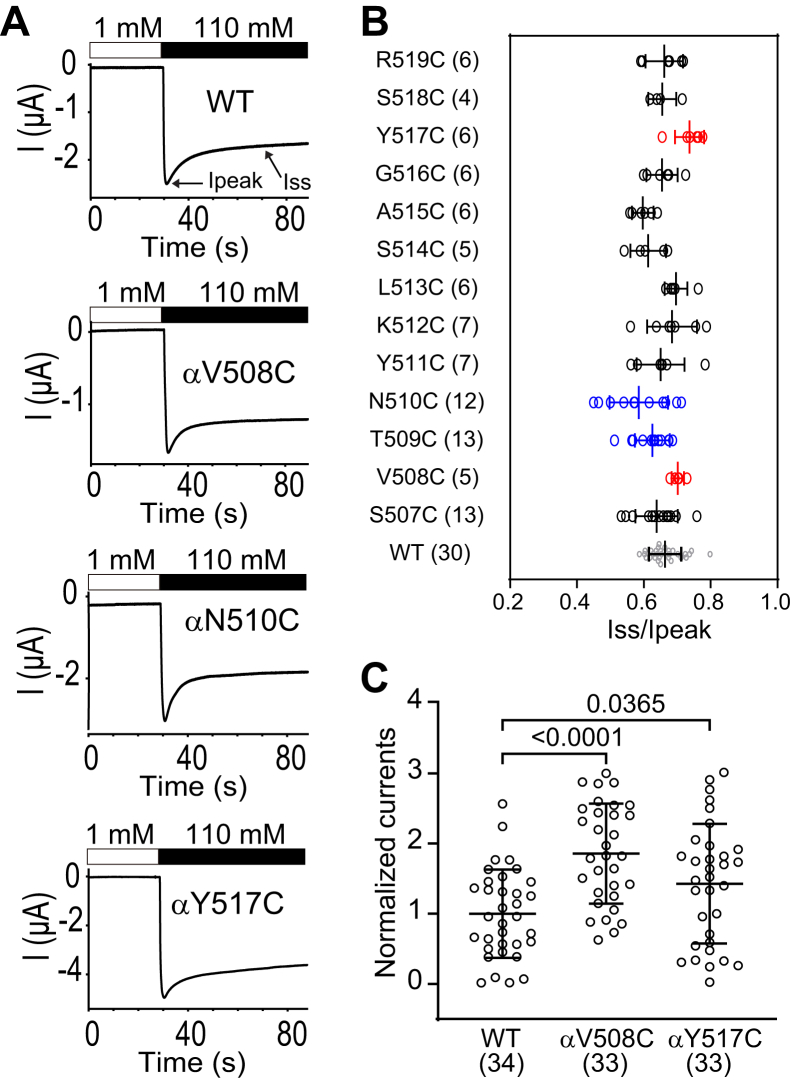
**Selected α subunit β10 Cys substitutions alter the Na**^**+**^**self-inhibition response**. Na^+^ self-inhibition responses were examined as described in the Experimental procedures. Oocytes expressing wildtype and mutant α subunit and wildtype β and γ subunits were perfused with bath solutions containing a 1 mM or a 110 mM Na^+^, as indicated by the open and filled bars in (*A*), while clamped at -100 mV. *A*, representative recordings showing the Na^+^ self-inhibition responses in wildtype and selected mutants. *B*, dot plots of Iss/Ipeak representing the magnitude of Na^+^ self-inhibition. Ipeak and Iss are peak and steady state currents, respectively, as depicted in *top panel* in (*A*). Bars are mean ± S.D. Numbers of oocytes in each experiment are listed in parentheses. Wildtype mouse αβγ ENaCs were always examined and compared with mutant channels in the same batch of oocytes. Statistical significances between wildtype and mutant channels were determined in the same batch of oocytes by one-way ANOVA followed by Dunnett’s tests. The values for wildtype channels (WT, shown in *gray*) in (*B*) were from all batches of oocytes used in these experiments and are shown for display purpose only. Values that are not significantly different from that measured with wildtype ENaC are shown in *black*. Values that are significantly reduced or increased when compared with wildtype (*p* < 0.05) are shown in *blue* or *red*, respectively. *C*, dot plot of the normalized currents (individual amiloride-sensitive currents divided by the mean wildtype current). Bars are mean ± S.D. Numbers of oocytes in each experiment are listed in parentheses. The *p* values were from one-way ANOVA followed by Dunnett’s tests. ENaC, epithelial Na^+^ channel.

We examined the Na^+^ self-inhibition response of each α subunit β10 Cys mutant and compared it to the wildtype Na^+^ self-inhibition response. As shown in Figure 6, two mutations (αV508C and αY517C) reduced and two mutations (αT509C and αN510C) enhanced the Na^+^ self-inhibition response. The data suggest that these residues have a role in Na^+^ self-inhibition. As the magnitude of Na^+^ self-inhibition is correlated with open probability (23), mutant channels with a reduced Na^+^ self-inhibition response should display an elevated whole-cell ENaC current, reflecting an increase in channel open probability. Indeed, amiloride-sensitive currents in oocytes expressing αV508Cβγ and αY517Cβγ were significantly greater than that in oocytes expressing wildtype αβγ mouse ENaC (Fig. 6*C*). Interestingly, currents in oocytes expressing either of these mutant channels were not significantly altered by either MTSES or MTSET (Fig. 3).

### Cys residues introduced at the proximal part of the *β*10 strand of the *β* subunit are functionally accessible to MTS reagents

To determine whether β10 strand residues in β and γ subunits exhibit a similar accessibility to MTS reagents, we introduced Cys mutations at four β and γ subunit β10 residues, homologous to the α subunit β10 residues where Cys substitutions either significantly reduced Na^+^ self-inhibition or rendered mutant channels MTS-sensitive.

**Figure 7:**
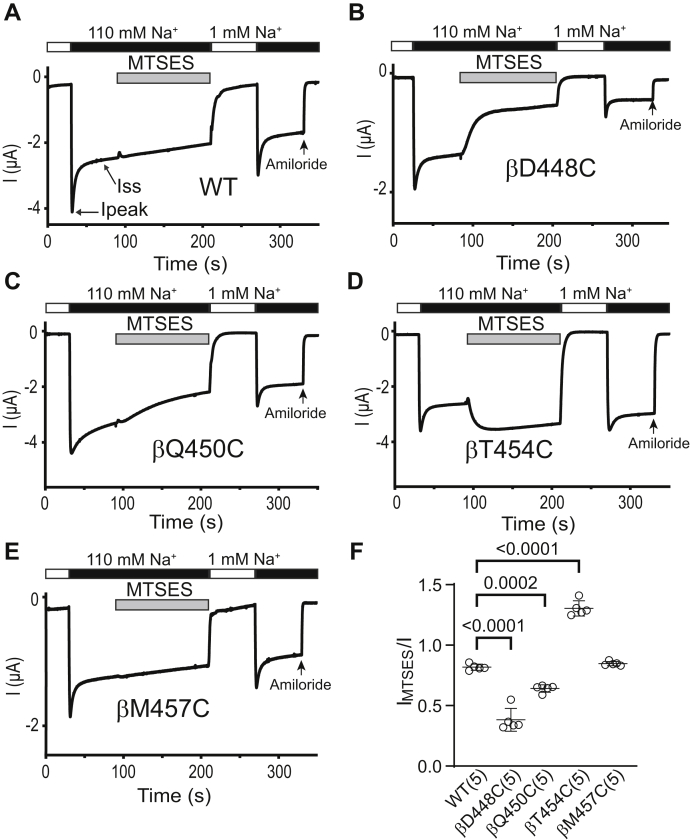
**Three proximal β subunit β10 residues are functionally accessible to MTSES.** Oocytes expressing wildtype or mutant β ENaC subunits together with wildtype α and γ subunits were clamped at a holding potential of −100 mV. Whole-cell Na^+^ currents were measured in oocytes perfused with solutions containing either a 1 mM (*white bar*) or a 110 mM Na^+^ (*black ba*r), as noted. *A*–*E*, MTSES (2 mM) was added to the bath, as indicated by the *gray* bar. Traces are representative of five experiments. Ipeak, peak current after switching from a 1 mM to a 110 mM Na^+^ bath solution. Iss, steady-state current at 40 s after Ipeak. The arrow indicates the addition of amiloride (10 μM) to the bath. *F*, I_MTSES_/I, determined as described in the Figure 3 legend. Mean ± S.D. (n = 5). The *p* values (above brackets) were from one-way ANOVA followed by Dunnett’s tests. ENaC, epithelial Na^+^ channel; MTSES, sodium (2-sulfonatoethyl) methanethiosulfonate.

**Figure 8:**
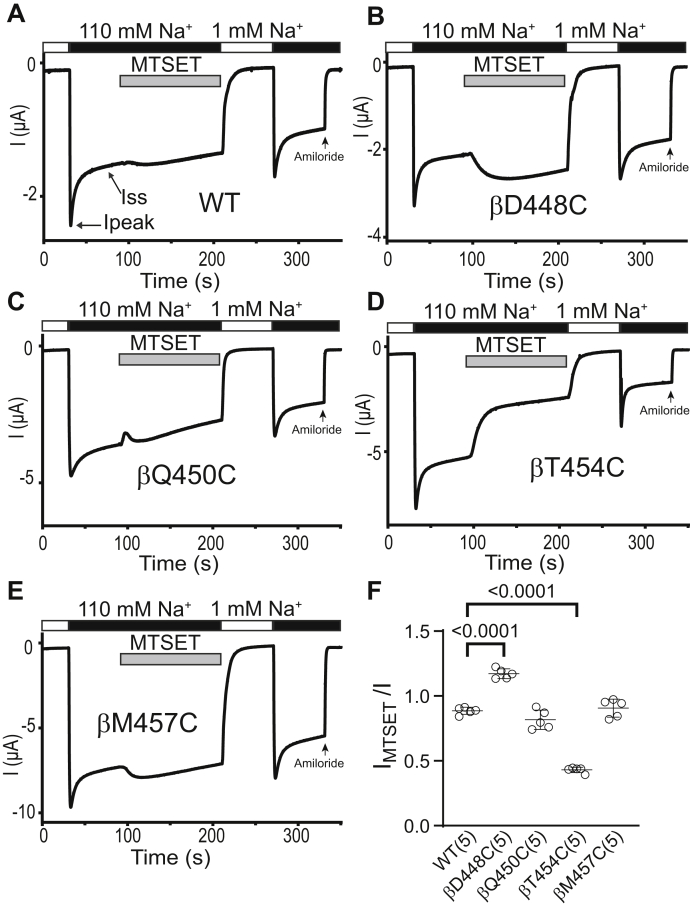
**Two proximal β subunit β10 residues are functionally accessible to MTSET.** Oocytes expressing wildtype or mutant β ENaC subunits together with wildtype α and γ subunits were clamped at a holding potential of −100 mV. Whole-cell Na^+^ currents were measured in oocytes perfused with solutions containing either a 1 mM (*white bar*) or a 110 mM Na^+^ (*black bar*), as noted. *A*–*E*, 1 mM MTSET was added to the bath, as indicated by the *gray bar*. Traces are representative of five identical experiments. Ipeak, peak current after switching from a 1 mM to a 110 mM Na^+^ bath solution. Iss, steady-state current at 40 s after Ipeak. The arrow indicates the addition of amiloride (10 μM) to the bath. *F*, I_MTSET_/I, determined as described in the Figure 3 legend. Mean ± S.D. (n = 5). The *p* values (above brackets) were from one-way ANOVA followed by Dunnett’s tests. ENaC, epithelial Na^+^ channel; MTSET, [2-(trimethylammonium) ethyl] methanethiosulfonate bromide.

As shown in Figure 7, MTSES significantly reduced currents in oocytes expressing αβD448Cγ (homologous to αV508Cβγ) and αβQ450Cγ (homologous to αN510Cβγ), while MTSES increased αβT454Cγ (homologous to αS514Cβγ) currents. It did not significantly change αβM457Cγ (homologous to αY517Cβγ) currents. MTSET inhibited αβT454Cγ currents but increased αβD448Cγ currents (Fig. 8). These results suggest that βD448C, βQ450C, and βT454C are functionally accessible to MTS reagents. Therefore, MTS accessibility is conserved between βQ450C and βT454C and their homologous sites in the α subunit (αN510C and αT514C). In contrast, while βD448C channels were responsive to MTS reagents, the homologous α subunit mutation (αV508C) is functionally inaccessible to MTS reagents (Figs. 3, 7 and 8).

**Figure 9:**
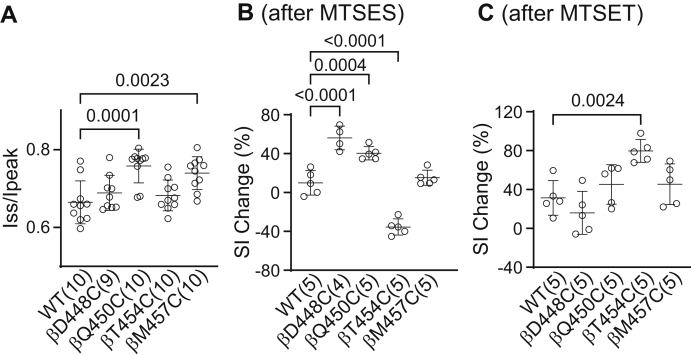
**Select β Cys substitutions alter the Na**^**+**^**self-inhibition response before and/or after MTS reagent treatment.** Na^+^ self-inhibition responses in oocytes expressing wildtype αβγ (WT) and mutant β plus wildtype α and γ ENaC subunits were examined prior to and after MTSES or MTSET treatment, as shown in Figures 7 and 8. *A*, dot plot of Iss/Ipeak. The Iss/Ipeak values were obtained from the experiments shown in Figure 7 (prior to MTSES) and 8 (prior to MTSET). Bars are mean ± S.D. (n=9–10). Iss and Ipeak are defined in Figures 7*A* and 8*A*. *B and C*, Na^+^ self-inhibition (SI) change (%) after MTSES (from experiments shown in Fig. 7) and MTSET (from experiments shown in Fig. 8). The values were calculated as described in the Figure 4 legend. Bars are mean ± S.D. (n = 4–5). All *p* values (above brackets) were from one-way ANOVA followed by Dunnett’s tests. ENaC, epithelial Na^+^ channel; MTSES, sodium (2-sulfonatoethyl) methanethiosulfonate; MTSET, [2-(trimethylammonium) ethyl] methanethiosulfonate bromide.

Compared to wildtype, αβQ450Cγ and αβM457Cγ showed a significantly reduced Na^+^ self-inhibition response (Figs. 7, 8 and 9*A*). The MTSES-induced current decrease in αβD448Cγ and αβQ450γ was associated with an enhanced Na^+^ self-inhibition response (Fig. 9*B*). Furthermore, the MTSES-induced increase in αβT454Cγ current was accompanied with a decreased Na^+^ self-inhibition response (Fig. 9*B*). Finally, the MTSET-induced current decrease in αβT454Cγ was followed by a significantly increased Na^+^ self-inhibition response (Fig. 9*C*). These results suggest that several β10 residues of β subunit have a role in Na^+^ self-inhibition response.

### Cys residues introduced at proximal part of the *β*10 strand of the *γ* subunit are functionally accessible to MTS reagents

**Figure 10:**
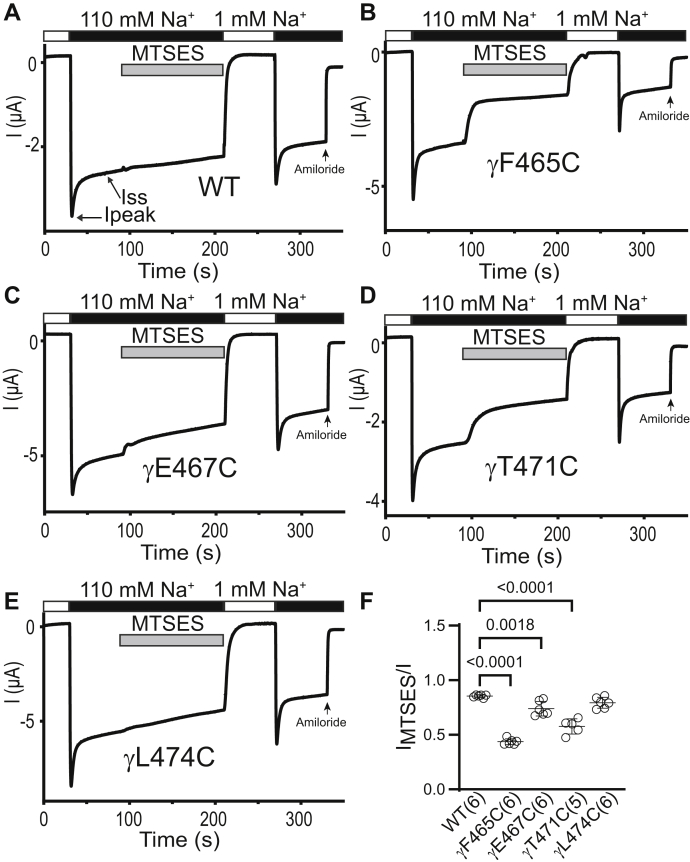
**Three proximal γ subunit β10 residues are functionally accessible to MTSES.** Oocytes expressing wildtype or mutant γ ENaC subunits together with wildtype α and β subunits were clamped at a holding potential of -100 mV. Whole-cell Na^+^ currents were measured in oocytes perfused with solutions containing either a 1 mM (*white bar*) or a 110 mM Na^+^ (*black bar*), as noted. *A*–*E*, 2 mM MTSES was added to the bath, as indicated by the *gray bar*. Traces are representative of five or six identical experiments. Ipeak, peak current after switching from a 1 mM to a 110 mM Na^+^ bath solution. Iss, steady-state current at 40 s after Ipeak. The arrow indicates the addition of amiloride (10 μM) to the bath. *F*, I_MTSES_/I, determined as described in the Figure 3 legend. Mean ± S.D. (n = 5–6). The *p* values (above brackets) were from one-way ANOVA followed by Dunnett’s tests. ENaC, epithelial Na^+^ channel; MTSES, sodium (2-sulfonatoethyl) methanethiosulfonate.

**Figure 11:**
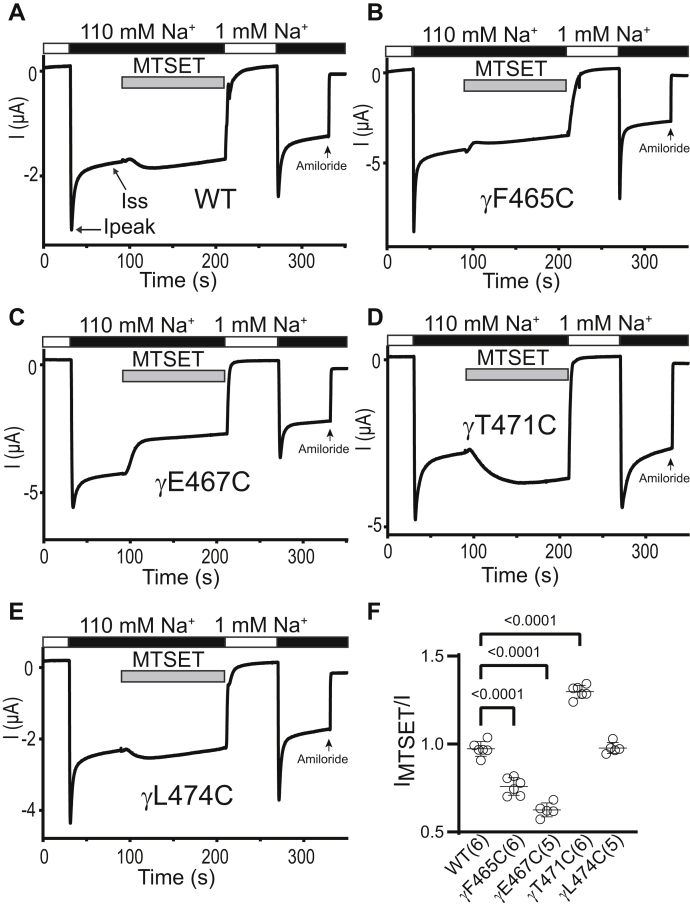
**Three proximal γ subunit β10 residues are functionally accessible to MTSET**. Oocytes expressing wildtype or mutant γ ENaC subunits together with wildtype α and β subunits were clamped at a holding potential of -100 mV. Whole-cell Na^+^ currents were measured in oocytes perfused with solutions containing either a 1 mM (*white bar*) or a 110 mM Na^+^ (*black bar*), as noted. *A*–*E*, 1 mM MTSET was added to the bath, as indicated by the *gray bar*. Traces are representative of five or six identical experiments. Ipeak, peak current after switching from a 1 mM to a 110 mM Na^+^ bath solution. Iss, steady-state current at 40 s after Ipeak. The arrow indicates the addition of amiloride (10 μM) to the bath. *F*, I_MTSET_/I, determined as described in the Figure 3 legend. Mean ± S.D. (n = 5–6). The *p* values (above brackets) were from one-way ANOVA followed by Dunnett’s tests. ENaC, epithelial Na^+^ channel; MTSET, [2-(trimethylammonium) ethyl] methanethiosulfonate bromide.

Among the four γ subunit mutants studied, αβγF465C (homologous to αV508Cβγ), αβγE467C (homologous to αN510Cβγ), and αβγT471C (homologous to αS514Cβγ) were inhibited by MTSES, whereas αβγL474C (homologous to αY517Cβγ) was not affected (Fig. 10). MTSET reduced αβγF465C and αβγE467C currents, while increasing αβγT471C currents. It did not affect αβγL474C currents (Fig. 11). These results suggest that γF465C, γE467C, and γT471C are functionally accessible to MTS reagents. Interestingly, MTS reactivity is conserved between three γ subunit residues (αβγF465C, αβγE467C, and αβγT471C) and their homologous β subunit residues (βD448C, βQ450C, and βT454C). While γE467C, γT471C, and homologous α subunit residues (αN510C and αT454C, respectively) are accessible to MTS reagents, we did not observe a consistent MTS response with γF465C (functionally responsive) and the homologous αV508C (functionally nonresponsive, Fig. 3).

**Figure 12:**
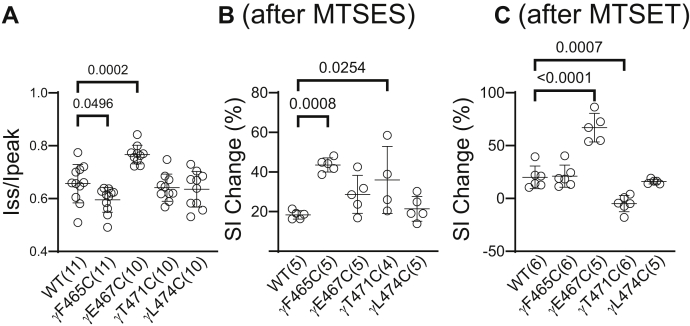
**Select γ Cys substitutions alter the Na**^**+**^**self-inhibition response before and/or after MTS reagents**. Na^+^ self-inhibition responses in oocytes expressing wildtype αβγ (WT) and mutant γ plus wildtype α and β ENaC subunits were examined prior to and after MTSES or MTSET treatment, as shown in Figures 10 and 11. *A*, dot plot of Iss/Ipeak. The Iss/Ipeak values were obtained from experiments shown in Figure 10 (prior to MTSES) and Figure 11 (prior to MTSET). Bars are mean ± S.D. (n = 10–11). Iss and Ipeak are depicted in Figures 10*A* and 11*A*. *B and C*, Na^+^ self-inhibition (SI) change (%) after MTSES (from experiments shown in Fig. 10) and MTSET (from experiments shown in Fig. 11), respectively. The values were calculated as described in the Figure 4 legend. Bars are mean ± S.D. (n = 4–6). All *p* values (above brackets) were from one-way ANOVA followed by Dunnett’s tests. ENaC, epithelial Na^+^ channel; MTSES, sodium (2-sulfonatoethyl) methanethiosulfonate; MTSET, [2-(trimethylammonium) ethyl] methanethiosulfonate bromide.

The Na^+^ self-inhibition response of αβγF465C (enhanced) and αβγE467C (reduced) differed from wildtype (Fig. 12*A*), while the Na^+^ self-inhibition response of the other γ subunit mutants was similar to wildtype. Following the inhibitory effect of MTSES, the Na^+^ self-inhibition response of αβγF465C and αβγT471C was enhanced compared to the response prior to MTSES. In contrast, the Na^+^ self-inhibition response of αβγE467C was not altered by MTSES (Fig. 12*B*). MTSET inhibited αβγE467C and enhanced the mutant’s Na^+^ self-inhibition response, whereas MTSET activated αβγT471C and reduced the mutant’s Na^+^ self-inhibition response (Fig. 12*C*). These data suggest that several γ subunit β10 residues have a role in Na^+^ self-inhibition.

### Cys residues introduced at select β10 sites are functionally accessible to Cd^2+^

**Figure 13:**
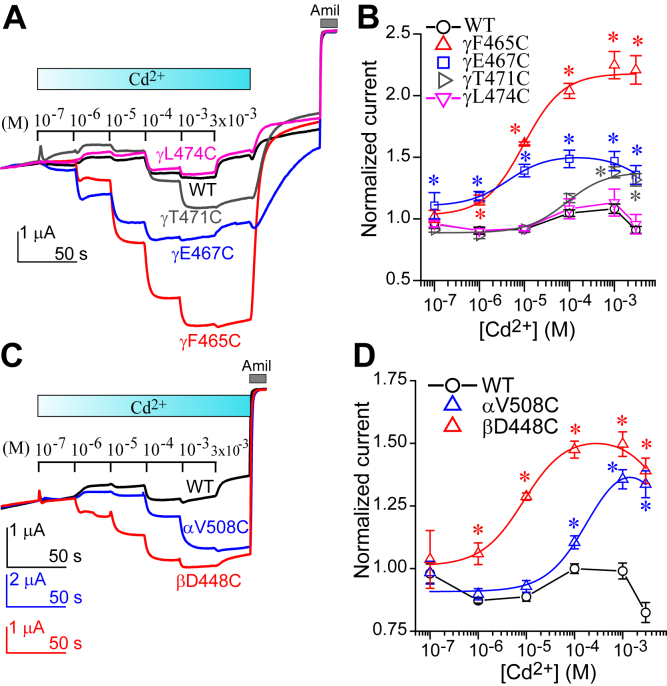
**Cd**^**2+**^**activates ENaCs with Cys substitutions in proximal β10 residues**. Effects of Cd^2+^ on whole-cell amiloride-sensitive currents in oocytes expressing wildtype and mutant ENaCs. Oocytes were perfused with a bath solution (NaCl-110) containing increasing concentrations of CdCl_2_, at a holding potential of -100 mV. *A*, representative recordings showing the effects of Cd^2+^ on wildtype αβγ, αβγF465C, αβγE467C, αβγT471C, and αβγL474C. Traces were superimposed to align the basal currents prior to Cd^2+^ treatment and the currents in the presence of 10 μM amiloride with approximately the same current and time scales. Cd^2+^ application is indicated by gradient cyan bars, and Cd^2+^ concentrations are shown above traces. *B*, dose responses of Cd^2+^ on amiloride-sensitive currents in oocytes expressing wildtype and the γ mutants. Normalized currents were the ratios of currents measured at the end of 30-s applications of Cd^2+^ and the basal currents measured immediately prior to 10^−7^ M Cd^2+^ application. Data are shown as mean ± S.D. (n = 5). Asterisks indicate that the values of mutant channels were significantly greater than that of wildtype channels (*p* < 0.01, one-way ANOVA followed by Dunnett’s tests). Values for αβγL474C did not differ from wildtype at all Cd^2+^ concentrations (*p* > 0.05). Smooth lines were from the best fit with the equation described in the Experimental procedures. The dose responses of Cd^2+^ on wildtype and αβγL474C channels were not fit successfully, and the data points are simply connected with straight lines. *C*, representative recordings showing the effects of Cd^2+^ on wildtype αβγ, αV508Cβγ, and αβD448Cγ. Traces were superimposed in a similar way to (*A*), but with distinct scales. *D*, dose responses of Cd^2+^ on amiloride-sensitive currents in oocytes expressing wildtype and the mutant channels. Asterisks indicate that the values of mutant channels were significantly greater than that of wildtype channels (*p* < 0.0001, one-way ANOVA followed by Dunnett’s tests). Smooth lines were from the best fit with the aforementioned equation. The dose responses of Cd^2+^ on wildtype channels were not fit successfully, and the data points are connected with black straight lines. EC_50_s and IC_50_s (±S.D.) are listed in Table 1. ENaC, epithelial Na^+^ channel.

We found that Cys residues introduced at multiple sites in the proximal parts of the β10 strands in all three subunits were accessible to MTS reagents. These MTS reagents are considered bulky, with estimated dimensions of 5 (short axis) by 10 (long axis) Å (32). We also examined accessibility and functional effects of a smaller, thiophilic cation, Cd^2+^ (10^−7^ M to 3 × 10^−3^ M) on currents in oocytes expressing wildtype channels or channels with a mutant γ subunit (Fig. 13*A*). The mutants αβγF465C and αβγE467C responded to both low and high concentrations of Cd^2+^ with significant and large increases in currents in a reversible manner, compared to wildtype. αβγT471C responded to high concentrations of Cd^2+^ with significant and moderate increases in currents. In contrast, wildtype and αβγL474C channels showed modest increases in current at high [Cd^2+^], which then fell at a [Cd^2+^] of 3 × 10^−3^ M. This modest activation was similar to what we previously observed with wildtype (33). The effects of Cd^2+^ were analyzed after normalizing amiloride-sensitive currents in the presence of Cd^2+^ to currents measured prior to Cd^2+^. Dose–response curves are shown in Figure 13*B*, and fitting parameters are summarized in Table 1. For αβγF465C, αβγE467C, and αβγT471C, data fit well with a two-site model, previously used to fit the response of ENaC to Zn^2+^ (34). The EC_50_s (±SD) for ENaC activation by Cd^2+^ (μM, n = 4–5) were 11 ± 2, 3 ± 1, and 124 ± 21 for αβγF465C, αβγE467C, and αβγT471C, respectively. We next examined the effects of Cd^2+^ on αV508Cβγ and αβD448Cγ channels (homologous to γF465C). Both mutants responded to Cd^2+^ with significant increases in currents (Fig. 13*C*), with estimated EC_50_s (μM, n = 4–5) of 253 ± 28 (αV508Cβγ) and 8 ± 1 (αβD448Cγ) (Fig. 13*D*). These results suggest that the binding of Cd^2+^ to substituted Cys residues at specific sites in the β10 strand of each subunit activates the channel.

**Table 1:** Fitting parameters for the dose responses of Cd^2+^ on mutant ENaCs

Channel	Imax	Imin	EC_50_ (μM)	EC_50_ (95% CI)	IC_50_ (mM)	R^2^	n
**αV508C**	1.5 ± 0.02	0.9 ± 0.03	253 ± 28	219–287	8.7 ± 2.7	0.93 ± 0.07	5
**βD448C**	1.5 ± 0.04	1.0 ± 0.02	8 ± 1	7–9	9.4 ± 3.0	0.98 ± 0.03	4
**γF465C**	2.2 ± 0.11	1.0 ± 0.04	11 ± 2	8–13	N.D.	0.99 ± 0.01	5
**γE467C**	1.5 ± 0.06	1.0 ± 0.03	3 ± 1	1–5	7.4 ± 3.1	0.98 ± 0.01	4
**γT471C**	1.5 ± 0.04	0.9 ± 0.02	124 ± 21	98–151	7.6 ± 2.1	0.98 ± 0.01	5

Note: Values are mean ± S.D.

95% CI, confidence interval at 95% for EC_50_ (μM); R^2^, square of correlation coefficient; N.D., not determined.

### Potential pathways for MTS reagents to reach introduced Cys residues

**Figure 14:**
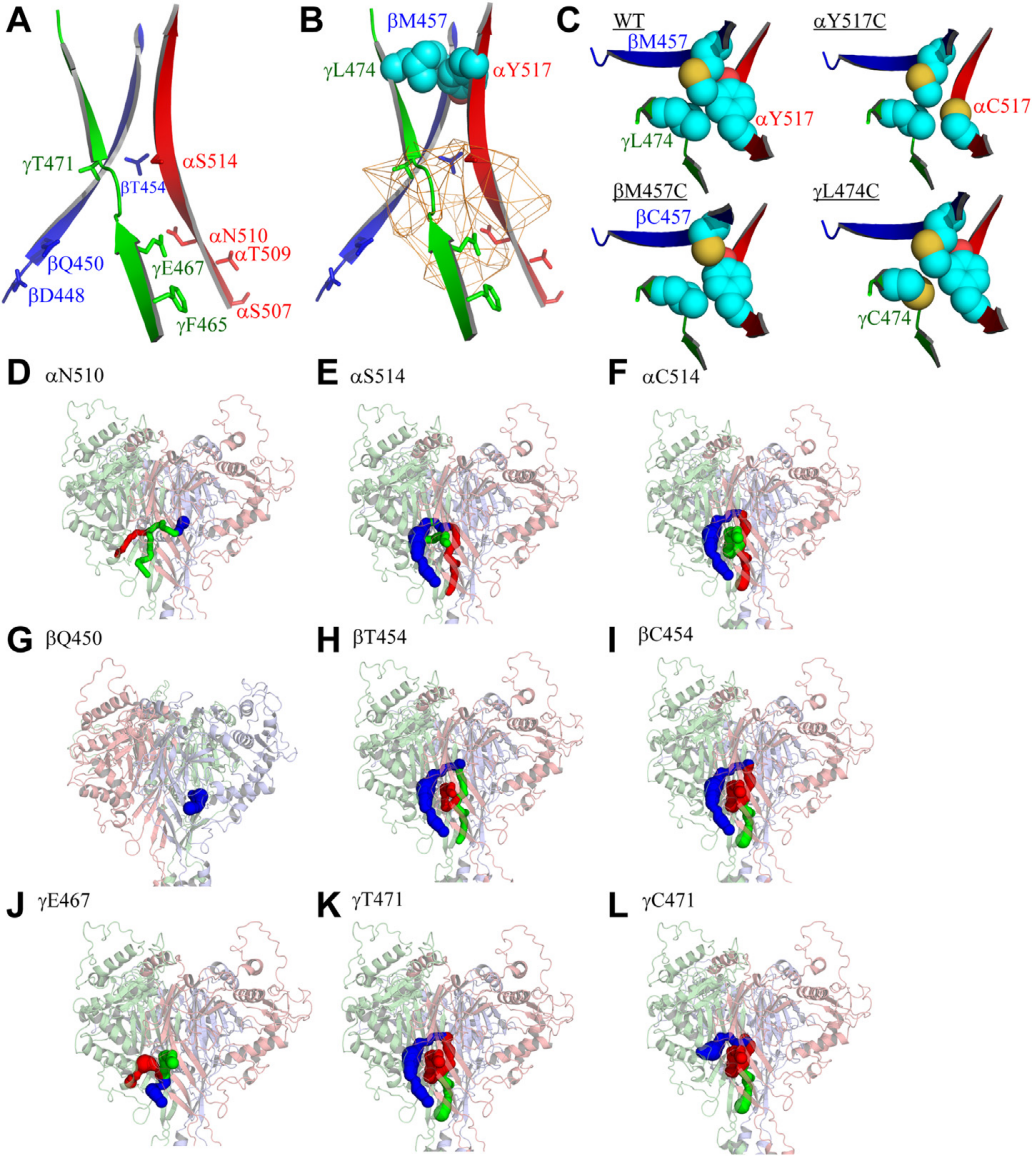
**Access tunnels in mouse ENaC**. *A*, a model of three mouse ENaC β10 strands (α subunit [*red*], β subunit [*blue*], and γ subunit [*green*]). Side chains of β10 residues that were accessible to MTSES or MTSET are shown as sticks. All 13 α Cys mutants were examined for MTS accessibility, whereas only four β Cys mutants (βD448C, βQ450C, βT454C, and βM457C) and γ Cys mutants (γF465C, γE467C, γT471C, and γL474C) were examined. *B*, a similar model to (A), with illustrated central vestibule and top seal. The central vestibule, shown as orange mesh, was calculated by CAVER Analyst 2.0 (35). Accessible residues are not labeled for clarity. Side chains of the three inaccessible residues (αY517, βM457, and γL474) that form a hydrophobic seal are shown as spheres with carbon in cyan, oxygen in red, and sulfur in yellow. *C*, top views of three β10 strands showing residues forming a hydrophobic seal at the central axis. Wildtype and mutant channels are labeled as WT, αY517C, βM457C, and γL474C. For mutants, only the mutated residue is labeled for clarity. Four panels were drawn with same rotation and scale to highlight the contact differences. The Cys substitutions were made in PyMol 2.4 using default rotamer. *D*-*L*, mouse ENaC models showing the top score tunnels identified by CAVER Web 1.1, with αN510 (D), αS514 (E), αC514 (F), βQ450 (G), βT454 (H), βC454 (I), γE467 (J), γT471 (K), or γC471 (L) as starting points. Mouse α, β, and γ subunits are shown in *light red*, *light blue*, and *light green*, respectively. The most prominent tunnel is shown in *blue*, while the second and third optimal tunnels are shown in *green* and *red*, respectively. The three tunnels for αN510 are overlapping. To optimally reveal the most promising *blue* tunnel in (*D*), the *green* and *red* tunnels are shown as thinner tunnels. There was only one tunnel identified with βQ450 as a starting point (*G*). For F, I, and L, a model with a single Cys substitution was used as the structural input. For others, a wildtype ENaC model was used. Tunnel search parameters are described in Experimental procedures. All models were visualized using PyMol 2.4 (75). ENaC, epithelial Na^+^ channel; MTSES, sodium (2-sulfonatoethyl) methanethiosulfonate; MTSET, [2-(trimethylammonium) ethyl] methanethiosulfonate bromide.

As the β10 strand is located within a three-fold axis of symmetry (Fig. 14, *A* and *B*) and most of its residues are not exposed to the surface, charged MTS molecules must travel to the introduced sulfhydryl groups through an aqueous tunnel. Inspection of a surface-rendered model of mouse ENaC did not reveal apparent openings to solvents. We used CAVER Analyst 2.0 (35) and CAVER Web 1.1 (36) to probe potential pathways for MTS reagents to access β10 residues. A search with a starting point at αN510 yielded three independent tunnels opening to the surface at various locations (Fig. 14*D*). The most promising aqueous tunnel (blue) with the largest bottleneck radius (1.4 Å) and shortest length (8 Å) opens to the surface at the junction of the α subunit palm, thumb, and β-ball domains (blue tunnel). A similar search starting at αS514 also revealed three tunnels (Fig. 14*E*). The most prominent tunnel leads from αS514 to the surface at the α (palm domain) and γ (thumb domain) interface (blue tunnel). Aqueous tunnels were also found to provide access to residues homologous to αN510 in the β (βQ450, Fig. 14*G*) and γ subunits (γE467, Fig. 14*J*), as well as access to residues homologous to αS514 in the β (βT454, Fig. 14*H*) and γ subunits (γT471, Fig. 14*K*). For αV508 and its homologous residues in the β and γ subunits (βD448 and γF465), their locations were too close to the surface to allow for reliable detection of tunnels. Using the same search parameters, no promising tunnel was identified for αY517 and its homologous residues in the β and γ subunits (βM457 and γL474), consistent with the functional insensitivity of their corresponding Cys mutants to MTS reagents. To determine whether substituted Cys affected the predicted tunnels, we repeated tunnel analyses on mutant ENaC models (generated by PyMol) containing the substituted Cys at sites selected as the starting point. These tunnels appear to be somewhat similar to tunnels based on the wildtype ENaC model. Representative tunnel models based on channels with specific Cys substitutions at three sites are shown in Figure 14*F*: (αC514), 14*I* (βC454), and 14*L* (γC471).

## Discussion

We used scanning Cys mutagenesis to explore the functional accessibility of β10 residues in the α subunit and whether MTS modifications affected channel gating as reflected in changes in the Na^+^ self-inhibition response. At least four of the first eight Cys substituted residues in the proximal aspect of α subunit β10 (αS507C, αT509C, αN510C, αS514C) were found to be functionally accessible to an MTS reagent, based on a large and significant change in current (Figs. 2 and 3). In contrast, none of the five Cys substituted residues in the distal aspect of β10 (αA515C-αR519C) responded to an MTS reagent with a significant change in current (Fig. 3). These observations are consistent with the concept that the proximal aspect of β10 within the palm domain contributes to the solvent accessible central vestibule, analogous to ASIC1 (6, 20). In addition, MTS reagents significantly altered the Na^+^ self-inhibition response in mutants that showed a strong response to these reagents, suggesting that MTS modifications of these introduced Cys residues within the α subunit β10 strand affected ENaC gating. Our study also showed that Cys mutations at multiple sites within β10 significantly altered the Na^+^ self-inhibition response.

In contrast to the proximal part of the β10 strand, all five sites with Cys introduced distal to αS514 were not functionally modified by either MTSES or MTSET. Our results clearly show differences in the functional accessibility of MTS reagents between the proximal and distal parts of β10 (Fig. 14*A*). However, we cannot rule out the possibility that sites in the distal aspect of β10 were chemically modified by MTS reagents. Our observations are consistent with the notion that distal palm domain provides a structural scaffold, while the proximal palm enables dynamic changes in conformations associated with channel gating (7, 20). The resolved structure of ASIC1 predicts a central aqueous pore/tunnel within the extracellular and transmembrane domains (37, 38). Our accessibility data and the tunnel computations do not support the notion of permeant ions entering the ENaC pore through an opening at the most distal aspects of the palm domain, unlike the long pore architecture found in pentameric acetylcholine receptors (39). Recent studies suggest that such a central pathway is unlikely to be an ion permeation pathway in P2X receptor channels (40, 41), which share a similar architecture with ASIC1 (42). Permeant cations and small pore blockers (*i.e.*, amiloride) likely gain access to the pore through the fenestrations formed at subunit interfaces near the transmembrane domain (6, 7). MTS reagents probably do not gain access to the introduced Cys residues in β10 strand through the same fenestrations, as these are only open to the vestibule within the pore, which is separated from the central vestibule by a net of lower palm domain β1 and β12 strands in resolved structures of ASIC1 and ENaC (7, 9, 10).

We also examined selected β10 residues in the β and γ subunits, focusing on sites homologous to α subunit β10 residues where channels with Cys substitutions were responsive to MTS reagents or where the Na^+^ self-inhibition response was altered. As expected, channels with Cys substitutions at sites homologous to αN510C (βQ450C and γE467C) and homologous to αS514C (βT454C and γT471) were functionally accessible to MTS reagents, whereas channels with distal β10 Cys substitutions homologous to αY517C (βM457C and γL474C) were unresponsive to these reagents (Figs. 7, 8, 10 and 11). While channels with an αV508C mutation did not respond to MTS reagents (Fig. 3), channels with Cys substitutions at homologous sites in the β and γ subunits (βD448C and γF465C) were functionally accessible to both MTS reagents (Figs. 7, 8, 10 and 11). Our observations regarding functional MTS accessible α, β, and γ subunit β10 residues are illustrated in Figure 14*A* and Table 2. It is interesting to note that not all accessible residues point their side chains to the central vestibule in the resolved ENaC structure (9, 10). Structural studies of ASIC1 (7, 20, 43) suggest that the proximal part of the β10 strand has a dynamic conformation and that side chain orientations are not static. The functional effects of MTS reagents do not follow a pattern where Cys substitutions at every other residue in the β10 strand are functionally modified by an MTS reagent, suggesting the aqueous accessibility is not limited to the central vestibule. The effects of MTS reagents on several mutants (αY511C and αK512C) were quite modest (Fig. 3) and were not included among the accessible residues in Figure 14*A*.

**Table 2:** Summary of changes in the Na^+^ self-inhibition response and response to MTS reagent treatment of ENaCs with Cys substitutions

Channel	S-I	MTSES	MTSET	MTSES *versus* MTSET	Accessible
**αY517C**	↓	−	−	−	No
**βM457C**	↓	−	−	−	No
**γL474C**	−	−	−	−	No
					
**αS514C**	−	↓↓	↓↓	Similar	Yes
**βT454C**	−	↑↑	↓↓	Opposite	Yes
**γT471C**	−	↓↓	↑	Opposite	Yes
					
**αN510C**	↑	↑↑	↓	Opposite	Yes
**βQ450C**	↓	↓	−	−	Yes
**γE467C**	↓	↓	↓↓	Similar	Yes
					
**αV508C**	↓	−	−	−	No
**βD448C**	−	↓↓	↑	Opposite	Yes
**γF465C**	↑	↓↓	↓	Similar	Yes

Channels with substitutions at homologous sites within ENaC subunits are clustered.

Note: S-I, Na^+^ self-inhibition: upward arrow, increased (or enhanced) Na^+^ self-inhibition; downward arrow, decreased (or blunted) Na^+^ self-inhibition; -, no significant change. MTSES and MTSET: upward arrow, increased current in response to the MTS reagent; downward arrow, decreased current in response to the MTS reagent; -, no significant change. Double arrows indicate greater changes than single arrows. MTSES *versus* MTSET: Similar, similar effect; Opposite, opposite effect; -, no comparison. Accessibility: No, no effect from both MTSES and MTSET; Yes, increase and/or decrease current in response to either MTSES or MTSET.

ENaC, epithelial Na^+^ channel; MTSES, sodium (2-sulfonatoethyl) methanethiosulfonate; MTSET, [2-(trimethylammonium) ethyl] methanethiosulfonate bromide.

The results from experiments on the homologous β10 residues in the three ENaC subunits are summarized in Table 2 and revealed several patterns regarding MTS-induced effects on mutant channels. First, channels with Cys substitutions that were responsive to MTSES were also responsive to MTSET, with the exception of βQ450C. Second, the effects of MTS reagents on channel activity (*i.e.*, increase or decrease) were dependent on the charge of the reagent for some mutants (αN510C, βD448C, βT454C, and γT471C). The effects of MTS reagents on other mutants (αS514C, γE467C, and γF465C) were not charge dependent. While both reagents inhibited both γF465C and γE467C mutants, the effect of MTSES was much greater than that of MTSET on the γF465C mutant and effect of MTSET was much larger than that of MTSES on the γE467C mutant (Figs. 10 and 11, Table 2). There are several charged residues in the β10 strands and nearby strands which may have a role on how mutant channels respond to charged MTS reagents. For example, γE467 in β10 is close to αR407 (β9 strand, 2.5 Å), well within the distance for salt bridge (44). MTSET modification of γE467C would not allow a salt bridge with αR407, consistent with the strong inhibition of αβγE467C activity in response to MTSET (Fig. 11*C*). In contrast, MTSES had a small effect on αβγE467C channel activity (Fig. 10*C*). Third, at homologous sites within the three ENaC subunits, the effects of both MTSES and MTSET were often subunit dependent. Functional ENaC subunit dependence has been well documented in many studies (12, 33, 45, 46, 47, 48, 49, 50, 51, 52, 53) and are consistent with the notion that homologous sites in ENaC subunits often have asymmetric functions (46, 47, 48). MTS reagents likely changed channel activity by altering ENaC gating, as suggested by their relatively rapid effect and significant change in the Na^+^ self-inhibition response induced by MTS modification. A lack of activation of αN510CβS518Kγ by MTSES (Fig. 5) also supports this notion.

The transition metal Cd^2+^ has been used to identify functional sites in ion channels (54). We examined whether ENaCs with Cys substitutions at specific sites in β10 responded to Cd^2+^ with a change in activity. Channels with Cys mutants at the three sites in the γ subunit that were MTS accessible (γF465C, γE467C, and γT471C) were also activated by external Cd^2+^ (Fig. 13, *A* and *B*). The most robust activation by Cd^2+^ was seen with the αβγF465C mutant. Channels with Cys substitutions at homologous sites in the α and β subunits (αV508C and βD448C) were also activated by Cd^2+^ (Fig. 13, *C* and *D*). The EC_50_ of Cd^2+^ activation varied among the mutants that were studied (Fig. 13). The Cd^2+^ EC_50_ is dependent on the numbers and types of side chains that form a Cd^2+^-binding site (54). ENaC mutants with EC_50_s of less than 12 μM (γF465C, γE467C, and βD448C) likely have a Cd^2+^-binding site that includes the introduced sulfhydryl group as well as nearby residues that coordinate Cd^2+^ binding (*i.e.*, Asp, Glu, and His). Coordinated Cd^2+^ binding is likely not present in ENaC mutants with higher EC_50_s (γT471C [124 μM] and αV508C [253 μM]) (54). These observations support the notion that these modified β10 residues have functionally important roles in ENaC gating. While most Cys mutants examined resulted in Cd^2+^-dependent activation, we saw either no effect or variable effects on channel activity in response to MTSES or MTSET (see Table 2). While the reason for these distinct effects Cd^2+^ and MTS reagents on ENaC activity is not clear, we speculate that the addition of different charged moieties with different sizes (Cd^2+^ *versus* MTSES and MTSET) likely perturbs local structures in distinct ways to alter channel activity. For example, the resolved ENaC structure shows a ring of polar residues, including αN510, βQ450, and γE467, which project their side chains to the central vestibule and contact multiple polar side chains from palm domains of the same or neighboring subunit (*i.e.*, γE467 may form a salt bridge with R407 within β9 strand of the α subunit). It is not surprising that modifying an introducing Cys residue with a bulky side chain at one of these sites will either enhance or disrupt interactions that affect channel structure and activity. In addition, Cd^2+^ coordination by an introduced sulfhydryl group and other native groups may strengthen polar interactions favoring an open state. Cd^2+^ binding might induce a collapsed central vestibule, favoring an open channel state (described in the later part of the article).

To further explore the aqueous accessibility of the proximal aspect of the β10 strands in the three ENaC subunits, we used CAVER Analyst 2.0 ((35)) and CAVER Web v1.1 (36) to identify extracellular aqueous tunnels in our mouse ENaC model. Potential tunnels were detected using empiric search parameters when αN510, αS514, or their homologous sites in the β or γ subunits were chosen as starting points (Fig. 14). The narrowest (bottleneck) radii of these tunnels were 1.4 to 1.9 Å, smaller than the 2.5 Å radius of MTSES or MTSET (32). Given the dynamic nature of protein structures, we predict that these tunnels allow the MTS reagents and Cd^2+^ to reach their targets. Only one tunnel was detected using βQ450 as starting point. The tunnel was similar to the most promising tunnel for αN510. Interestingly, the most promising tunnels (in blue) for αS514, βT454, and γT471 were also similar. No tunnels were detected for αY517, βM457, and γL474 using the same search parameters, consistent with the observations that their corresponding Cys mutants did not respond to MTS reagents. For all sites studied, no tunnels transiting along the three-fold symmetry axis from the top of the extracellular domain were identified.

Several β10 mutations significantly altered the channel’s Na^+^ self-inhibition response. Among the 13 α mutants studied, αY517C and αV508C exhibited reduced Na^+^ self-inhibition and greater amiloride-sensitive currents, compared to wildtype (Fig. 6). These observations are consistent with an increased channel open probability, as predicted from the close correlation between the magnitude of Na^+^ self-inhibition and channel open probability (23). Interestingly, the gain-of-function human ENaC variant αV481M, homologous to mouse αV508, had a reduced Na^+^ self-inhibition response (55). Two βENaC mutations (βQ450C and βM457C) and one γENaC mutation (γE467C) exhibited a significantly reduced Na^+^ self-inhibition response (Figs. 9*A* and 12*A*), while three mutants (αT509C, αN510C, and γF465C) had an enhanced Na^+^ self-inhibition response (Figs. 6 and 12*A*). Our results suggest that these eight sites with β10 residues have a role in the mechanism of Na^+^ self-inhibition, likely influencing allosteric transitions that occur following Na^+^ binding. These β10 residues are not in the vicinity of the previously defined α subunit Na^+^-binding site (10, 17).

ENaC structures (9, 10) and our structural model suggest that hydrophobic contacts from αY517, βM457, and γL474 within the distal part of β10 strands seal the central axis at a site distal to the solvent-accessible central vestibule (Fig. 14*B*). This hydrophobic seal likely supports the scaffold formed by the distal parts of β10 strands. Our models predict that the αY517C mutation creates a separation between the αY517C, βM457, and γL474 (Fig. 14*C*). The βM457C mutation also creates a separation between βM457C and γL474, though it retains close contact with αY517. In contrast, γL474C appears to be less disruptive. The observations are consistent with the suppressed Na^+^ self-inhibition response observed with αY517C and βM457C, while γL474C did not affect Na^+^ self-inhibition (Figs. 6, 9*A* and 12*A*). Given the proximity of this hydrophobic seal to a hydrophobic patch in the knuckle domain implicated in Na^+^ self-inhibition response (47), we speculate that the hydrophobic seal and the knuckle domain hydrophobic patch function as an integral nonpolar anchor within the extracellular domain. Stability of this anchor may facilitate conformational changes of other flexible parts in response to extracellular Na^+^ or other channel modulators. Consistent with this notion, mutations within the β10 hydrophobic seal, the hydrophobic knuckle patch, or deletion of the knuckle domain in the α subunit reduce or eliminate Na^+^ self-inhibition (Figs. 6 and 9*A* and (47)).

Our results regarding the effects of β10 Cys substitutions on Na^+^ self-inhibition and the response to MTS reagents and Cd^2+^ suggest that the β10 strands in ENaC subunits, which reside at the three-fold symmetry axis, have functional roles in ENaC gating. The β10 strands interface with thumb and knuckle domains, which also have roles in modulating ENaC gating (47, 48). Moreover, β10 within the α subunit is flanked by residues (αC506 and αW520, Fig. 1*C*) where introduced mutations alter ENaC gating. Mouse αC506 is homologous to human αC479, where a Cys-to-Arg mutation was found in a pair of siblings with a Liddle syndrome phenotype (56). We previously showed that αC506 and αC421 form a disulfide bond, and disruption of this bond affects Na^+^ self-inhibition (49). αW520 is homologous to human αW493, a site where a gain-of-function variant (αW493R) was identified (57, 58). Our results suggest that nonsynonymous variants of human ENaC at specific β10 sites are likely to alter ENaC activity. In addition, the β10 strand may convey conformational changes from peripheral ENaC helical domains to structures that directly control ENaC gating. The central locations of the β10 strands allow for extensive intrasubunit and intersubunit contacts that likely have roles in modulating ENaC gating. Intersubunit interactions between palm and thumb domains have been demonstrated to contribute to ENaC gating and regulation by extracellular Cl^-^ and Cu^2+^ (52, 53, 59). Previous work examining the functional effects of varying length bifunctional cross-linkers also highlights the importance of intersubunit interactions (53).

The proximal aspect of the ASIC palm domains has been implicated in channel activation and desensitization (7, 19, 20, 43, 60, 61, 62, 63). ENaCs do not undergo desensitization, and it is unclear whether ENaCs Na^+^ self-inhibition response resembles ASIC desensitization. Functionally relevant acidic residues in the proximal palm domains of ASICs are not present in ENaC subunits, consistent with distinct mechanisms regulating channel gating. Within ASIC1a, unique acidic residues E80 (β1), E417 (β12), E412 (β11-β12 linker), and E374 (β10) contribute to protonation and Ba^2+^-binding sites (6, 64). The proposed Ba^2+^-binding sites in the central vestibule of ASIC1a, involving Q277, E374, E412, and E417 (64), are not present in ENaC.

Various roles of the central vestibule in ASIC1 have been proposed, including serving as a cation reservoir, as well as roles in channel desensitization and modulator binding (7, 20, 43, 64, 65). We speculate that the major function of the central vestibule of ENaC is to facilitate conformational changes associated with channel opening and closing. Consistent with this notion, certain ions or small molecules may modulate ENaC gating by binding to residues within the central vestibule and altering channel gating. Extracellular ions such as Na^+^, H^+^, Cl^-^, and certain divalent cations and small molecules are important ENaC-gating modulators (1, 2, 11, 12, 13, 14, 34, 50, 52, 53, 59, 66, 67, 68, 69, 70, 71). The mechanisms by which these ions or small molecules alter channel gating are not fully understood. Binding within the central vestibule provides a plausible pathway for certain modulators to influence ENaC gating, similar to what has been suggested for ASICs (64). Small molecules may gain access to the central vestibule through the routes identified in our study. Although binding sites for Na^+^, H^+^, and Cl^-^ have been reported outside of the central vestibule (10, 17, 52, 72), a βENaC Val (V348) was identified as a central vestibule residue that facilitates ENaC activation by small molecules (S3969 and 8-(4-chlorophenylthio)adenosine 3′,5′-cyclic monophosphate) (69, 73). In addition, mouse αN510 is homologous to guinea pig αI481, a site implicated in ENaC activation by 8-(4-chlorophenylthio)adenosine 3′,5′-cyclic monophosphate (68).

Previous studies suggest that ASIC1 palm domain β sheets lining the central vestibule undergo conformational changes in association with channel gating (7, 20). During proton activation, ASIC1 undergoes a slight contraction of the central vestibule (7, 64). We speculate that the ENaC central vestibule exhibits similar conformational changes in association with gating. A potential coordination of Cd^2+^ by multiple ligands from introduced sulfhydryl groups (*i.e.*, γE467C, γF465C, or βD448C) and native groups is consistent with a reduction of the central vestibule space in association with channel activation. Clearly additional studies are needed to ascertain changes in the structure of ENaC’s central vestibule during gating transitions.

In summary, our study identified solvent-accessible residues within the proximal parts of the palm domain β10 strands of the three ENaC subunits. Furthermore, several β10 residues were found to have roles in modulating ENaC gating.

## Experimental procedures

### Site-directed mutagenesis

Point mutations in mouse ENaC α, β, and γ subunits were introduced using QuickChange Ⅱ XL site-directed mutagenesis kit (Agilent). Mutations were confirmed by DNA sequencing conducted in the Genomics Research Core of University of Pittsburgh. Linearized DNAs following restriction enzyme digestion were purified by GeneJET PCR Purification Kit (Thermo Fisher Scientific). Wildtype and mutant mouse ENaC cRNAs were generated *via* T3 RNA polymerase (mMESSAGE mMACHINE T3 Kit, Invitrogen) and purified by RNeasy MinElute Cleanup Kit (QIAGEN). Concentrations of plasmid DNAs and cRNAs were quantified by spectrophotometry.

### ENaC expression

Oocytes from female *Xenopus laevis* were harvested according to a protocol approved by the University of Pittsburgh’s Institutional Animal Care and Use Committee and treated with type II collagenase (Sigma-Aldrich). cRNA (0.5 or 1 ng) of each mouse ENaC subunit (wildtype or mutant α, wildtype or mutant β, and wildtype or mutant γ) was injected into stage V or stage VI oocytes, and oocytes were incubated at 18 ^°^C in a modified Barth’s solution: 88 mM NaCl, 1 mM KCl, 2.4 mM NaHCO_3_, 15 mM Hepes, 0.3 mM Ca (NO_3_)_2_, 0.41 mM CaCl_2_, 0.82 mM MgSO_4_, 10 μg/ml streptomycin sulfate, 100 μg/ml gentamycin sulfate, and 10 μg/ml sodium penicillin, pH 7.4.

### Two-electrode voltage clamp

Two-electrode voltage clamp studies were performed at room temperature (20–24 °C) 1 to 2 days after injection using Axoclamp 900A Computer-Controlled Microelectrode Amplifier and DigiData 1440A controlled by pClamp 10.4 (Molecular Devices). Oocytes were placed in a chamber with constant flow (∼5 ml/min). Glass pipettes filled with 3 M KCl were inserted into oocytes, and the intracellular potential was clamped at −100 mV.

### Na^+^ self-inhibition

The Na^+^ self-inhibition response was assessed as previously reported (12). Oocytes expressing wildtype or mutant channels were perfused with a 1 mM Na^+^ bath solution (NaCl-1: 1 mM NaCl, 109 mM N-methyl-D-glucamine, 2 mM KCl, 2 mM CaCl_2_, and 10 mM Hepes, pH 7.4) for 60 s and switched abruptly to a 110 mM Na^+^ bath solution (NaCl-110: 110 mM NaCl, 2 mM KCl, 2 mM CaCl_2_, and 10 mM Hepes, pH7.4). Currents were recorded by a two-electrode voltage clamp. After 60 s of NaCl-110 perfusion, 10 μM amiloride was applied to determine the amiloride-sensitive current. Upon the change from NaCl-1 to NaCl-110, currents rapidly increased to a peak (Ipeak) and slowly declined to a steady-state level (Iss, measured 40 s after Ipeak).

### Effects of MTSET and MTSES

MTSES (Toronto Research Chemicals) powder was freshly dissolved in NaCl-110 at a concentration of 2 mM and used for experiments within 2 h. MTSET (Toronto Research Chemicals) solution was prepared in NaCl-110 at a concentration of 1 mM and used immediately. MTSES and MTSET solutions were perfused for 2 min, when currents were stable. Amiloride-sensitive Na^+^ currents prior to and after MTSES or MTSET application were measured as I and I_MTSES_ or I_MTSET_, respectively.

### Effects of Cd^2+^

The effects of Cd^2+^ on selected mutant and wildtype channels were examined by monitoring the current changes following 30 s applications of increasing concentrations of CdCl_2_, in the range of 10^-7^ to 3 x 10^-3^ M. A CdCl_2_ stock (1 M) was prepared by dissolving CdCl_2_ powder in deionized water and diluted to various concentrations of CdCl_2_ in the NaCl-110 bath solution prior to use. Oocytes were perfused with NaCl-110 containing 10 μM amiloride after 3 mM CdCl_2_ or after washout with NaCl-110 for 1 min in order to measure the amiloride-insensitive component of the whole-cell current. The Cd^2+^ dose response was analyzed using a two-site equation I=(Imax-Imin)(C/(C+ EC_50_))(IC_50_/(C+IC_50_))+Imin, as previously described (34). The I, Imax, and Imin are the observed, maximal, and minimal values of the normalized currents, respectively, while C, EC_50_, and IC_50_ are the [Cd^2+^] used in our studies, the [Cd^2+^] that achieved 50% channel activation, and the [Cd^2+^] that achieved 50% channel inhibition, respectively. Chelators were not added to the NaCl-110 solution. Concentrations of CdCl_2_ added to the bath solution were used to represent [Cd^2+^], which was not the free Cd^2+^ concentrations that would be calculated using a chelator. Nonlinear regression fitting was performed with Origin Pro 2018 (OriginLab Corporation).

### Molecular modeling

A structural model of mouse ENaCs was generated with SWISS-MODEL (22). Templates were automatically searched from input amino acid sequences Q61180 (SCNNA_MOUSE), Q9WU38 (SCNNB_MOUSE), and Q9WU39 (SCNNG_MOUSE). Eight templates with the highest quality, originated in PDB 6BQN (9) but with varying lengths, were automatically selected for modeling. A template originated in the structure of human ENaC (PDB 6WTH (10)) was deemed less suitable for modelling than the selected eight templates. Models were built based on target-template alignment using ProMod3 (22) and ranked according to sequence identity and other parameters. The final model was chosen based on its highest sequence identity (84.4) and quaternary structure quality estimate (0.85 (74)). Models were illustrated using PyMol 2.4 (75). Solvent access pathways were analyzed by CAVER Analyst 2.0 (35) and CAVER Web 1.1 (36). Search configuration parameters were empirically optimized after a series of trials with varying parameter inputs using CAVER Analyst 2.0. The numbers of tunnels were minimized by using the most stringent search parameters which still allowed identification of at least one tunnel with a meaningful length (>5 Å). Final lists of tunnels for each mutant mouse ENaC were obtained from CAVER Web 1.1 using following search parameters: probe radius, 1.4; shell radius, 5; shell depth, 4; and clustering threshold, 5. Tunnel analyses were performed using wildtype and mutant ENaC models as structural inputs. The mutant models containing single Cys substitution were generated by PyMol 2.4.

### Statistical analyses

Data are presented as mean ± S.D. Given the inherent batch-to-batch variation in expression levels in *Xenopus* oocytes, all comparisons between mutant and wildtype channels were done in the same batches of oocytes. Significance between two groups was determined by Student’s *t* test with Welch’s correction and for multiple groups by one-way ANOVA followed by Dunnett’s correction. p＜0.05 was considered statistically significant. All significance comparisons and normality tests (Shapiro–Wilk test) were performed using GraphPad Prism 9.

## Data availability

All data are contained within the manuscript.

## Conflict of interests

The authors declare that they have no conflicts of interest with the contents of this article.
